# The Facile Synthesis of Branch-Trunk Ag Hierarchical Nanostructures and Their Applications for High-Performance H_2_O_2_ Electrochemical Sensors

**DOI:** 10.3390/s17122896

**Published:** 2017-12-13

**Authors:** Yan Zhang, Meiqiong Chen, Zhiquan Cai, Min Zhang, Peng Liu, Faliang Cheng

**Affiliations:** 1 Department of City and Environmental Sciences, College of Science and Technology of Dongguan of City College, Dongguan 523419, China; zhangyan@ccdgut.edu.cn (Y.Z.); chenmq@ccdgut.edu.cn (M.C.); caizq@ccdgut.edu.cn (Z.C.); 2 Guangdong Engineering and Technology Research Center for Advanced Nanomaterials, School of Environment and Civil Engineering, Dongguan University of Technology, Dongguan 523808, China; mindear@dgut.edu.cn (M.Z.); liupeng@dgut.edu.cn (P.L.)

**Keywords:** branch-truck Ag, hierarchical nanostructure, H_2_O_2_, electrochemical sensor

## Abstract

A novel branch-trunk Ag hierarchical nanostructure was synthesized via a galvanic replacement reaction combined with microwave-assisted synthesis using Te nanowire as a sacrificial template. The Te nanowire was synthesized via a hydrothermal process. We further investigated the potential application of the obtained hierarchical nanostructures in electrochemical sensor analysis. The results showed that the as-prepared sensor exhibited a wide linear range with 0.05 µM to 1.925 mM (R = 0.998) and the detection limit was estimated to be 0.013 µM (S/N = 3). These results indicate the branch-truck Ag hierarchical nanostructures are an excellent candidate material for sensing applications.

## 1. Introduction

Recently, three dimensional (3D) hierarchical nanostructure have provoked considerable interest due to their rich architectures, distinct properties and various novel applications [1,2,3,4]. Controlled synthesis of hierarchical nanostructure is highly desirable, owing to their structure merits. Among the myriad of possible nanostructures, branch-truck nanostructures have been studied in recent years because a great deal of branch nanostructure on a nanowire backbone could provide an even higher surface-to-volume ratio compared to single nanowires. For example, Her et al. proposed a simple two-step thermal vapor transport process to prepare brush-like p-Te/SnO_2_ hierarchical nanostructures, which were used to detect NH_3_ and exhibited excellent catalytic behavior [5]. Khoang et al. designed a branch-truck hierarchical SnO_2_/ZnO nanostructure by combining a thermal evaporation method and a hydrothermal approach. These SnO_2_/ZnO hierarchical nanostructures were systematically investigated for ethanol-sensing. These results revealed that the electrochemical sensors based on hierarchical nanostructures showed high sensibility and selectivity [6].

H_2_O_2_ is widely used in fields such as organic synthesis [7], environmental detection [8], biology [9], clinical [10], pharmaceutical [11], food production [12], sterilization [13] and so on [14,15]. In particular, it plays a role of considerable importance in the metabolic processes of organisms [16]. Therefore, it is critically important to develop an accurate, fast and reliable method for the detection of H_2_O_2_. Compared with other analytical methods for detecting of H_2_O_2_ [17,18,19,20,21], electrochemical sensors have become one of the hottest topics due to their higher sensitivity, simpler operation and lower cost [22,23,24] However, direct reduction of H_2_O_2_ at bare electrodes show poor sensitivity, low selectivity and poor reproducibility. Because of the high electrochemically active area as well as excellent electronic transfer capability, metal nanomaterials have been tried out as sensing materials to modify the electrode surface for developing H_2_O_2_ sensors with high sensitivity and good selectivity.

As a typical noble nanomaterial, Ag nanomaterial exhibits excellent conductivity as well as high catalytic properties. This was because nanoscale Ag show a rapid specific surface area increase and more atoms exposed on the surface make for increased active sites. In the catalytic decomposition of H_2_O_2_, Ag nanomaterials could accelerate the electron transfer rate, reduce the reaction energy and amplify the detection signal. For example, we have reported high sensitivity H_2_O_2_ sensors based on a perpendicular Ag nanowires array [25]. The catalytic properties of nanomaterials are closely related to their size, morphology and the types of composites. Therefore, it was important to prepare novel Ag nanostructures with superior function. Ag nanoparticles [26], nanowires [27], nanoplates [28], hollow hierarchical microspheres [29,30] have all been reported as sensing materials for developing H_2_O_2_ electrochemical sensors. However, to the best of our knowledge, up to now, no branch-truck Ag nanostructures have been reported for this purpose. Branch-trunk Ag hierarchical nanostructures provide a very high electrochemically active area and thereby lead to high detection sensitivity. They could also act as enhanced elements for effectively accelerating the electron transfer between the electrode and probe molecules, which would lead to a rapid current response and in the meantime reduce the overpotential of electrochemical reactions.

In this paper, we report the synthesis of branched-truck hierarchical nanostructures containing Ag nanowire trunks and Ag nanorod branches by a simple two-step synthesis pathway. High-quality single crystalline Te nanowire backbones were synthesized using a hydrothermal approach, whereas Ag nanorod branches were subsequently grown perpendicularly to the axis of Te nanowires via microwave-assisted synthesis (EG as solvent and reducing agent, PVP as polymer surfactant and CuCl_2_ as control agent). The as-prepared branched-truck hierarchical nanostructure-modified electrode was used as a novel high-performance H_2_O_2_ sensor. The experimental data illustrated that the branch-truck Ag hierarchical nanostructure-modified electrodes demonstrated excellent catalytic performance for H_2_O_2_ reduction, while detection with a wide linear range, good selectivity and long-term stability was achieved.

## 2. Experimental

### 2.1. Materials

K_3_Fe(CN)_6_, K_4_Fe(CN)_6_, ammonium hydroxide, acetone, hydrazine (50%), KH_2_PO_4_, K_2_HPO_4_ were purchased from Tianjin Damao Chemical Factory (Tianjin, China). PVP [poly-(*N*-vinyl-2-pyrrolidone)] was obtained from Sigma-Aldrich (Darmstadt, Germany), AgNO_3_, Na_2_TeO_3_ and H_2_O_2_ was obtained from Xiya Reagent (Chengdu, China). All the chemicals were of analytical grade. Distilled water was used in the experiments. 

### 2.2. Apparatus

The electrochemical experiments were performed using a CHI660D electrochemical workstation (Chenhua, Shanghai, China). A standard three-electrode system (Ag branch-trunk hierarchical nanostructures modified electrode as work electrodes, Pt as the counter electrode and Ag/AgCl (saturated KCl) as reference electrode) was used in all the experiments. Scanning electron microscopy (SEM) analyses were performed using a 6701F instrument (JEOL, Tokyo, Japan). X-ray diffraction (XRD) analysis was carried out on an Ultima IV X-ray diffractometer (Rigaku, Tokyo, Japan). A microwave oven with 10–500 W power was used.

### 2.3. Synthesis of Branch-Truck Ag Hierarchical Nanostructures

Te nanowires were synthesized according to the reference [31]. Briefly, 1.0 g PVP and 0.0922 g of sodium tellurite were dissolved in 35 mL of double-distilled water to form a homogeneous solution under vigorous magnetic stirring at room temperature. Then 1.65 mL of hydrazine hydrate and 3.35 mL of aqueous ammonia solution were added into the previous solution. The final solution was transferred into a Teflon-lined stainless steel autoclave (50 mL in total volume), which was closed and maintained at 180 °C for 4 h, and then the sample was allowed to cool to room temperature. 110 mL of acetone was added into the final solution to precipitate the product, which was then centrifuged and washed with double-distilled water and absolute ethanol several times, respectively. Synthesis of branch-truck Ag hierarchical nanostructure: in brief, 15 mL of 1 mM CuCl_2_/EG was prepared firstly. Then, 1.27 mg of Te nanowires and 1 g PVP was added into the above solution, followed by the addition of 5 mL of 20 mM AgNO_3_/EG solution. The resulting solution was then transferred to a microwave for heating reaction at (temperature). After the reaction, the solution was cooled to room temperature. The final product was obtained, in order to remove the remaining CuCl_2_, EG and PVP, after the solution was centrifuged and washed in acetone, alcohol and deionized water at a speed of 9000 r/min for 5 min each, respectively.

### 2.4. Fabrication of Branch-Truck Ag Hierarchical Nanosturture Sensor

First, bare glass electrode (GCE) was polished on suede with 1.0 and 0.3 μm Y-Al_2_O_3_ powder, respectively. The electrodes were washed with ethanol and double distilled water for 20 min, and then the performance of bare glass electrodes were detected in K_3_Fe(CN)_6_/K_4_Fe(CN)_6_. 1 mg branch-truck Ag hierarchical nanostructures were dispersed in 1 mL Nafion solution (1%) for 10 min. The suspension solution was dispersed onto a GCE surface with a microsyringe and dried at room temperature. 

## 3. Results and Discussion

### 3.1. Characterization

Te nanowires were used as substrate and templates to prepare the branch-truck Ag hierarchical nanostructures. Figure S1 shows the SEM image of the as-prepared Te nanowires with a diameter of several hundred nanometers. XRD patterns of the Te nanowires were showed in Figure S2. All peaks can be indexed with the standard JCPDS card no. 36–1452 which confirms the hexagonal phase of tellurium nanowires. The peaks observed at 2θ values of 23.08°, 27.72°, 38.36°, 40.52°, 43.44°, 45.98°, 46.92°, 49.63°, 56.82°, 62.78°, 63.93°, 65.72°, 67.64°, 71.92°, and 75.24°are assigned to the diffractions from (100), (101), (102), (110), (111), (003), (200), (201), (202), (113), (210), (211), (104), (212) and (304) planes, respectively. Figure S3 show cyclic voltammograms of Te nanowires modified electrode in PBS solution with scan rate of 50 mVs^−1^. A pair of sharp redox peaks at +0.264 V and −0.74 V are due to the self-redox of Te nanowires [32].

### 3.2. The Influence of Synthesis Condition:

**Figure 1:**
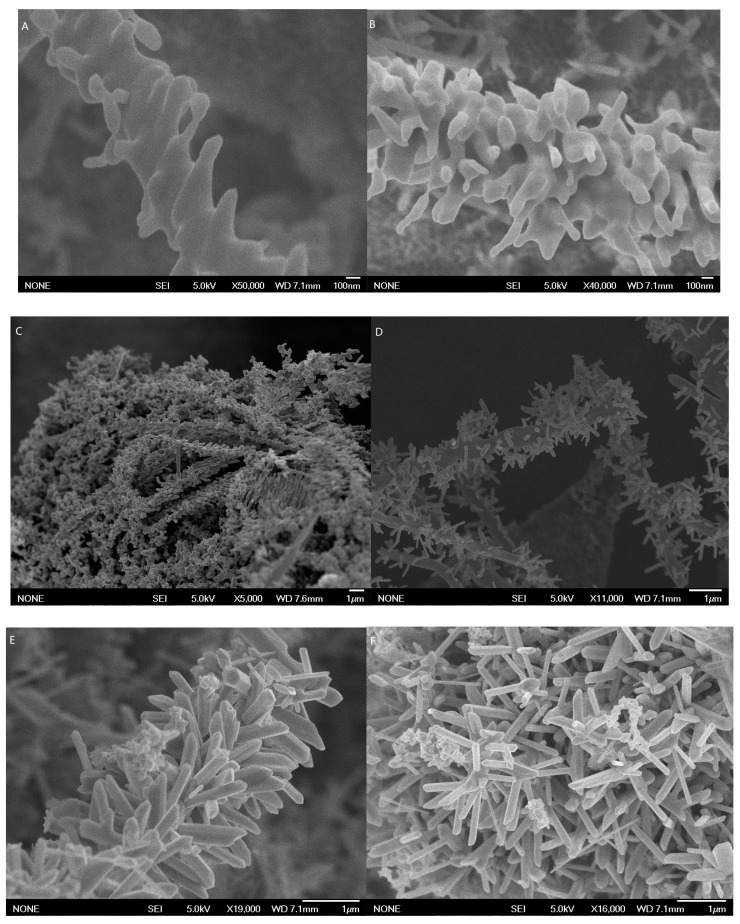
Typical SEM images of the branch-trunk Ag hierarchical nanostructures of different reaction time. (**A**,**B**) 30 s ; (**C**,**D**) 60 s; (**E**,**F**) 90 s.

When Te nanowires were used as precursor and template, the hierarchical Ag nanostructure was obtained via microwave-assisted synthesis by adjusting the synthetic parameters. In the presence of PVP and CuCl_2,_ the microwave power was set at 300 W and the reaction time was set at 30 s, Te-Ag core shell nanowire backbones were found to be fully covered by Ag thin layers with abundant flake-like Ag nanostructures (Figure 1A,B). 

When the reaction time was prolonged to 60 s, complete nanorods with about 50–80 nm in width and 100–300 nm in length grew perpendicularly on the surface of the nanowires (Figure 1C,D). We further increased the reaction time to 90 s, and the branched Ag nanostructure was still growing in size. Ag nanorods eventually completely covered the nanowire backbones to form branch-stem Ag hierarchical nanostructures with an average diameter of ca. 100 nm and lengths of several hundreds of nanometers (Figure 1E,F). When the microwave power was set at 500 W and the microwave time was set at 90 s, vertical nanoplates will spring out of the Ag nanowire backbones to form Ag nanoplates on the surface of Te nanowire structures (Figure S4A,B). Vertical nanoplates can be generated either sparsely or densely by increasing the number of platelets at higher growth temperatures and lower pressures [33]. In the absence of PVP and CuCl_2_, when the microwave power was set at 300 W and the microwave time was set at 90 s, Ag nanoparticles with irregular sizes and shapes were found to be generated on the surface of Te nanowires whereas no nanorods were observed on the Ag nanowires backbone (Figure S5A,B). This is because PVP, as a surfactant, will direct the growth of Ag to the nanorod structure form [34,35,36]. 

**Figure 2:**
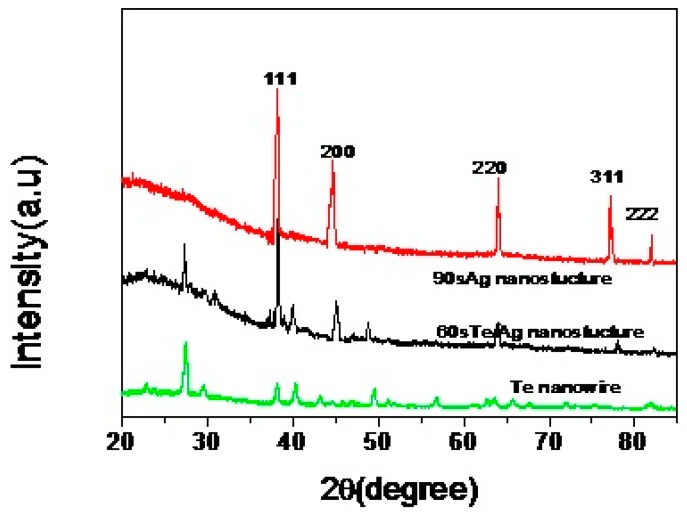
XRD patterns of branch-trunk Ag hierarchical nanostructures generated at different reaction time.

**Figure 3:**
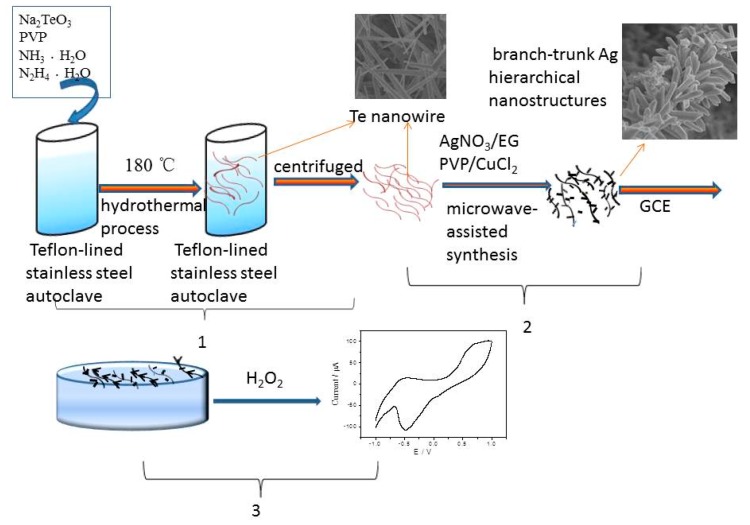
Schematic illustration of the formation mechanism of branch-trunk Ag hierarchical nanostructures and branch-trunk Ag hierarchical nanostructure-modified GCE used for detecting H_2_O_2_.

The XRD patterns of as-synthesized branch-stem Ag hierarchical nanostructures with various growth times are shown in Figure 2. The XRD patterns of 60 s branch-stem Ag hierarchical nanostructures is the mixture of diffraction peak of silver and tellurium. For comparison, the peaks of 90 s branch-stem Ag hierarchical nanostructures occurring at 38.14°, 44.32°, 64.43°, 77.56°, and 81.54° were attributed to diffractions from the (111), (200), (220), (311), and (222) crystal planes, which were consistent well with a face-centered cube of Ag. No diffraction peaks corresponding to Te were found, which suggested that the Te nanowire backbone have been completely replaced with Ag. The schematic illustration of the growth mechanism of Ag branches on the Ag nanowire backbones by microwave-assisted synthesis was shown in Figure 3. 

An enormous amount of silver nanoparticles was synthesized via microwave-assisted synthesis. Ag cations replace Te in Te nanowires to form Ag nanoparticles at Te nanowires surface via a galvanic displacement reaction. The reaction between Te nanowires and Ag^+^ can be formulated as follows:

Ag^+^ + Te + 3H_2_O = Ag + TeO_3_^2−^ + 6H^+^ (1)

As the galvanic replacement reaction proceeded, the Ag nanoparticles would grow along Ag crystal seeds on the surface of the Ag nanowire to form nanospikes and nanoflakes. As the reaction continued, the Ag nanospikes and nanoflakes increased in size gradually and formed nanorods. Finally, high-quality Ag nanorods were produced and attached to the Ag nanowires.

**Figure 4:**
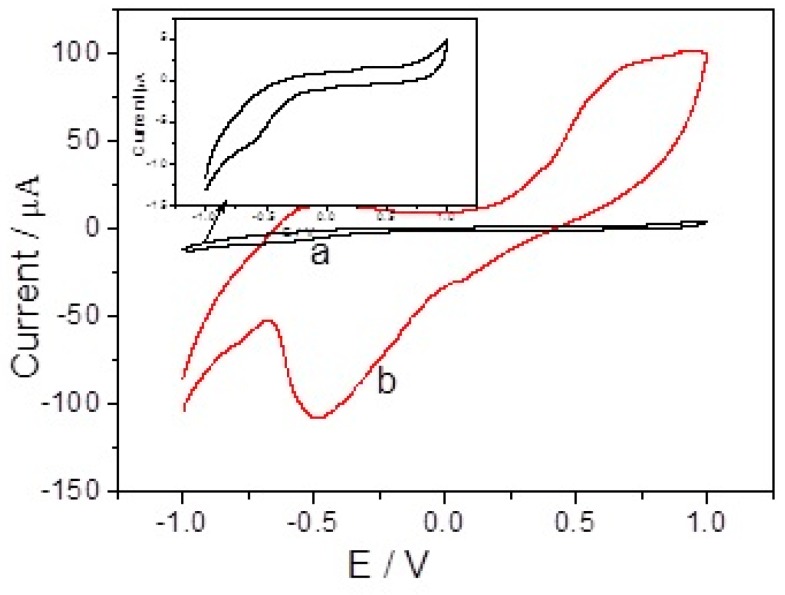
CVs of bare electrode (a) and branch-trunk Ag hierarchical nanostructures/GCE (b) in 0.1 M PBS (pH = 7.4) in the presence of 0.5 mM H_2_O_2_. Scan rate: 50 mVs^−1^.

**Figure 5:**
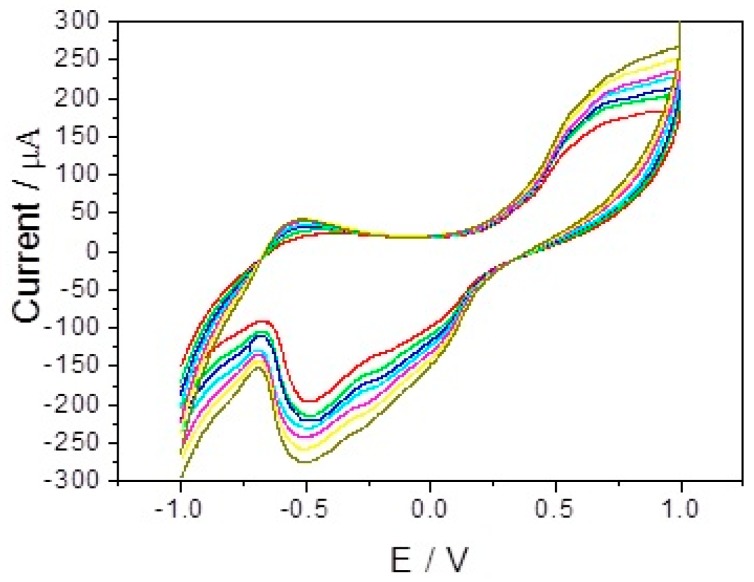
CVs of branch-trunk Ag hierarchical nanostructures/GCE in 0.1 M PBS (pH = 7.4) containing, 2 mM, 3 mM, 4 mM, 5 mM, 6 mM, 7 mM, 8 mM H_2_O_2_. Scan rate: 50 mVs^−1^.

Figure 4 depicts cyclic voltammograms (CVs) of bare GCE (a) and branch-truck Ag hierarchical nanostructures/GCE (b) in 0.1 M PBS (pH = 7.4) in the presence of 0.5 mM H_2_O_2_ with scan rate of 50 mVs^−1^. A strong peak at about −0.48 V, attributed to the reduction of H_2_O_2_, was seen on branch-truck Ag hierarchical nanostructures modified GCE whereas a very small reduction peak was obtained at −0.61 V for H_2_O_2_ at bare GCE. The insert of Figure 4 shows the cyclic voltammogram of bare GCE in 0.1 M PBS (pH = 7.4) in the presence of 0.5 mM H_2_O_2_ with scan rate of 50 mVs^−1^. Compared with bare GCE, a positively shifted peak potential of 110 mV was observed on the branch-truck Ag hierarchical nanostructures modified/GCE. There was an increase in current by increasing H_2_O_2_ concentration. When H_2_O_2_ concentration was increased from 2 mM to 8 mM, an increase reduction current was observed (Figure 5). Thus, branch-truck Ag hierarchical nanostructures could greatly reduce the overpotential and increase the catalytic current toward the reduction of H_2_O_2_, which was important for improving the high sensitive and selective detection for H_2_O_2_.

Direct electrochemical reduction of H_2_O_2_ at ordinary solid electrodes shows low electrode kinetics and high overpotential. Based on the available literature, the mechanism of H_2_O_2_ electroreduction can be described as follows [37]:

OH (aq) + e^−^ ⇋ OH^−^ (2)

H_2_O_2_ + e^−^ ⇋ OH (aq) + OH^−^ (3)

2OH^−^ + 2H^+^ ⇋ 2 H_2_O (4)

In the case of silver-catalyzed decomposition of H_2_O_2_, the reaction turned more irreversible [37]:

(5) $$H_{2}O_{2}\;\overset{Ag}{\to }1/2\;O_{2}+H_{2}O$$

(6) $$O_{2}+2e^{-}+2H^{+}\;\to \;H_{2}O_{2}$$

The O_2_ produced in the catalytic reaction would induce detection signal on the electrode. According to some theories in the literature, the mechanism of the O_2_ reduction on the electrode as described below [38,39]:

O_2_ + e^−^ ⇋ [O^·−^] _(ads)_ (7)

[O^·−^] _(ads)_ + H_2_O ⇋ HO_2_^·^_(ads)_ +OH^−^ (8)

Then:

HO_2_·_(ads)_ +O_2_^·−^ ⇋ HO_2_^−^_(ads)_ +O_2_ (9)

or:

HO_2_^·^_(ads)_ +e^−^ ⇋ HO_2_^−^_(ads)_ (10)

**Figure 6:**
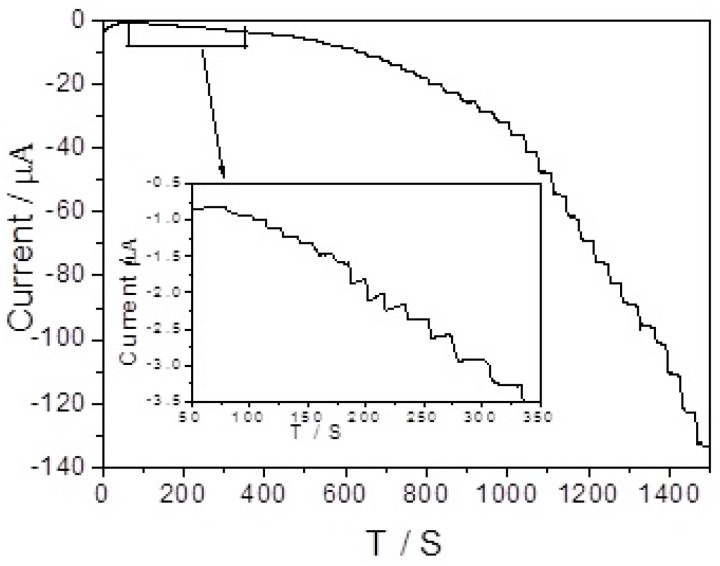
Amperometric response of the sensor for successive addition of H_2_O_2_ in N_2_-saturated 0.1 M PBS (pH = 7.4) solution. The applied potential is −0.5 V, (inset: a partial magnification of the current response toward a low concentration of H_2_O_2_ solution).

**Figure 7:**
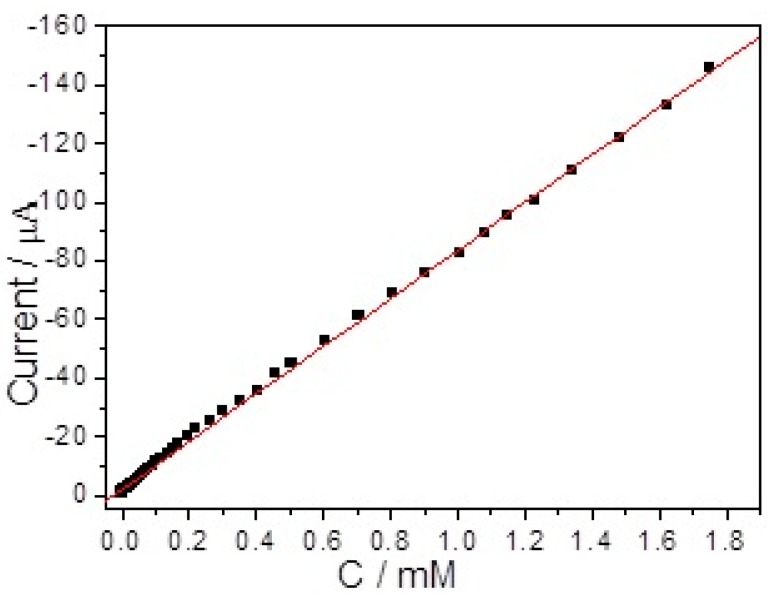
The calibration curve of the amperometric response.

Figure 6 shows the amperometric response of the branch-truck Ag hierarchical nanostructures/GCE with gradually additions of H_2_O_2_ to the PBS solution under stirring conditions. As the potential was set at −0.5 V, the branch-truck Ag hierarchical nanostructures/GCE responded rapidly and achieved steady-step current by adding H_2_O_2_ to the solution. The insert of Figure 6 shows the low concentration range. The corresponding linear calibration curve shown in the inset of Figure 7 reveals there were a good linear relationship between the reduction peak current and the H_2_O_2_ concentration with the range of 0.05 μM to 1.925 mM with a correlation coefficient of 0.998. The regression equation was I (μA) = −83.18 C − 2.1897 (C is the concentration of H_2_O_2_ in mM). The detection limit of H_2_O_2_ sensors was 0.013 μM (S/N = 3). 

The sensitivity was calculated to be 325.52 μA mM^−1^ cm^−2^. The as-fabricated H_2_O_2_ sensors were compared with those of Ag nanomaterials-based non-enzymatic H_2_O_2_ sensors in Table 1. The proposed method was better than the previous reports. To illustrate the feasibility in practical sample analysis of the proposed branch-truck Ag hierarchical nanostructures/GCE the determination of H_2_O_2_ in milk samples were studied. The real samples were diluted 10 times with water and diluted milk samples were streamed into five groups separately (S. No 1, S. No 2, S. No 3, S. No 4, S. No 5), and then different concentrations of H_2_O_2_ (0.01, 0.05, 0.10, 0.15, and 0.20 mM) were spiked into five groups (S. No 1, S. No 2, S. No 3, S. No 4, S. No 5) of practical samples. The chronoamperometric responses were recorded of the spiked concentration, measured concentration of H_2_O_2_, and relative standard deviation (RSD) (three repeated experiments). The results, listed in Table 2, showed good reliability and reproducibility for the branch-truck Ag hierarchical nanostructures/GCE.

**Figure 8:**
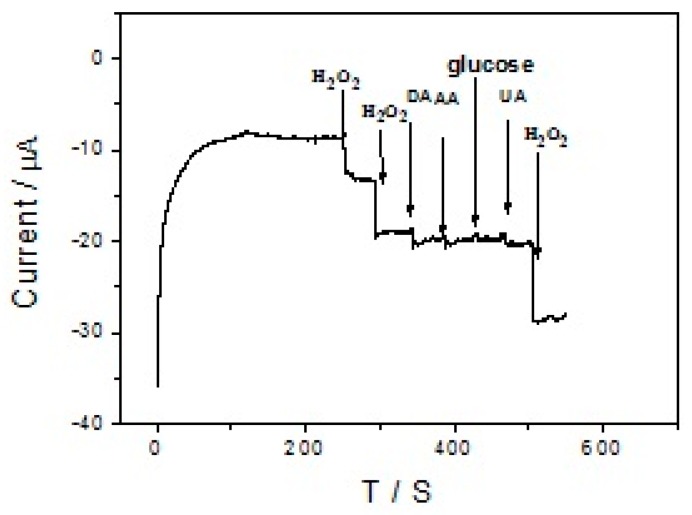
Amperometric curves of branch-trunk Ag hierarchical nanostructures/GCE for successive additions of H_2_O_2_ and different interfering substances in the 0.1 M PBS solution (pH 7.4). The applied potential is −0.5 V.

In practical samples analysis, some coexisting biomolecules can cause serious interference in the detection of a single component. Possible interfering species for detecting H_2_O_2_ such as dopamine (DA), ascorbic acid (AA), glucose and uric acid (UA) were investigated by amperometric response (Figure 8) with additions of 100 μL 1 mM H_2_O_2_, 1 mM H_2_O_2_, 10 mM AA, 10 mM DA, 10 mM UA and 10 mM glucose and 200 μL 1 mM H_2_O_2_. The branch-truck Ag hierarchical nanostructures/GCE showed a high sensitivity amperometric response to H_2_O_2_ whereas there is no obvious amperometric response for the interfering species AA, glucose and UA. Additionally, the interferent DA only produces a weak reduction current at −0.5 V. Therefore, at an applied potential of −0.5 V coexistint interferents do not interfere with the selective determination of H_2_O_2_.

**Figure 9:**
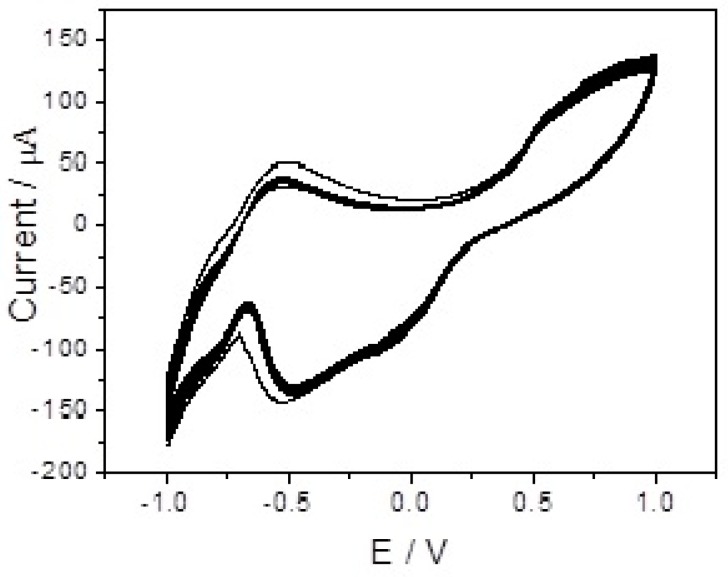
The repeatability of branch-trunk Ag hierarchical nanostructures/GCE in 1 mM H_2_O_2_ solution after 10 scanning cycles.

As important evaluation sensor parameters, the repeatability and stability of the H_2_O_2_ sensors were investigated. The branch-truck Ag hierarchical nanostructures modified electrodes were used to detect 1 mM H_2_O_2_ with the applied potential range of −1.0 V to 1.0 V. The catalytic current decreased to 90.5% after 10 scanning cycles, illustrating that the as-prepared sensor had highly reproducibility (Figure 9). Furthermore, the relative standard deviation (RSD) for five branch-truck Ag hierarchical nanostructures modified electrodes to detect the same 0.5 mΜ H_2_O_2_ solution was 4.1%, further indicating the good reproducibility of the branch-truck Ag hierarchical nanostructures-based H_2_O_2_ sensor. To study the stability of the sensor, the branch-truck Ag hierarchical nanostructures modified GCE was tested after 21 days, 90% of its initial current response was remained. These results show the sensor has satisfactory performance. 

## 4. Conclusions

Branch-truck Ag hierarchical nanostructures were synthesized via a combination of hydrothermal synthesis with microwave-assisted synthesis. The as-fabricated materials could be used as an excellent charge-transfer substrate for the electrocatalytic reduction of H_2_O_2_. The prepared electrochemical H_2_O_2_ sensor showed good catalytic performance with a wide linear range, low detection limit and good selectivity and reliability. Therefore, the sensor has potential application for the selective determination of H_2_O_2_ in real sample analysis.

## Figures and Tables

**Table 1:** Comparison of the fabricated sensor with other Ag material-based non-enzyme sensors for H_2_O_2_.

Electrode	Sensitivity (μA mM^−1^ cm^−2^)	Linear Range (μM)	LOD (μM)	Ref
Ag NPs/rGO/GCE	225	50–5000	10	[40]
Ag Y/CPE	650.7	20–5000	1.4	[41]
Ag NPs/rGO/GCE	---	10–6000	1.8	[42]
Ag-Au@Cu_2_O NWs/GCE	---	100–9000	1.1	[43]
AgNPs/SBA-16/GCE	816.6	20–8000	2.95	[44]
Ag@SiO_2_@Ag/GCE	56.07	5–24,000	1.7	[45]
AgNPs/X-CPE	60.6	20–11,760	9.1	[26]
Ag/boehmite NTs/rGO/GCE	80.14	0.5–10,000	0.17	[46]
branch-truck Ag HN/GCE	325.52	0.05–1925	0.013	our report

Where GCE—glassy carbon electrode, CPE—carbon paste electrode, Ag NPs—Ag nanoparticles, rGO—reduced grapheme oxide, Ag Y—Ag nanoparticles arrays, Ag-Au@Cu_2_O NWs—Cu_2_O nanocubes decorated by Ag-Au alloy nanoparticles, SBA-16—mesoporous SBA-16 nanoparticles, Ag@SiO_2_@Ag—Ag@SiO_2_@Ag, X—NaX nanozeolite, boehmite NTs—Ag decorated boehmite nanotubes, branch-truck Ag HN—branch-truck Ag hierarchical nanostructures.

**Table 2:** The practical sample analysis result of hydrogen peroxide for the branch-truck Ag hierarchical nanostructures/GCE in pH 7 PBS (three repeated experiments).

S. No	Added Concentration (mM)	Found Concentration (mM)	Recovery (%)	RSD (%)
1	0.0100	0.01012	101.2	3.1
2	0.0500	0.05005	100.5	1.6
3	0.1000	0.09987	99.87	3.2
4	0.1500	0.15015	100.1	5.6
5	0.2000	0.01996	99.8	1.8

Where S. No 1—diluted milk sample 1, S. No 2—diluted milk sample 2, S. No 3—diluted milk sample 3, S. No 4—diluted milk sample 4, S. No 5—diluted milk sample 5.

## Supplementary Materials

The following are available online at www.mdpi.com/1424-8220/17/12/2896/s1, Figure S1: Typical SEM images of Te nanowires, Figure S2: XRD patterns of Te nanowires, Figure S3: CVs of Te nanowires in 0.1 M PBS solution (pH = 7.4), Scan rate: 50 mVs^−1^, Figure S4: A and B SEM images of Ag nanoplates on nanowires with different magnification, Figure S5: A and B SEM images of Ag nanoparticle on nanowires with different magnification.
