# Supplementary material for: The Facile Synthesis of Branch-Trunk Ag Hierarchical Nanostructures and Their Applications for High-Performance H2O2 Electrochemical Sensors
Source: Sensors (Basel). 2017 Dec 13;17(12):2896. doi: 10.3390/s17122896 (PMC5751687; doi:10.3390/s17122896)
Supplement: Supplementary file 1 [file sensors-17-02896-s001.pdf]

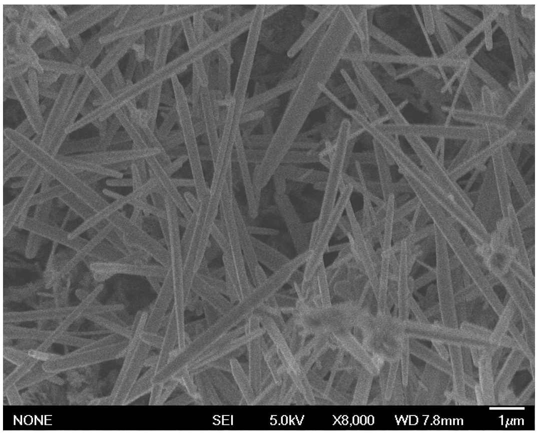

Figure S1. Typical SEM images of Te nanowires.

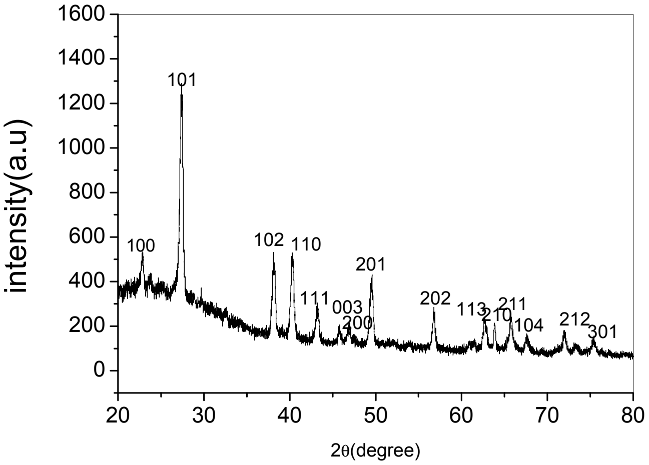

Figure S2. XRD patterns of Te nanowires.

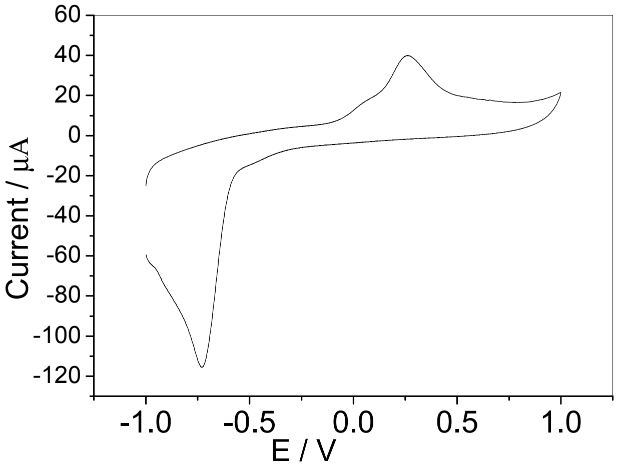

Figure S3. CVs of Te nanowires in0.1M PBS solution(pH=7.4), Scan rate: 50mVs^{-1}.

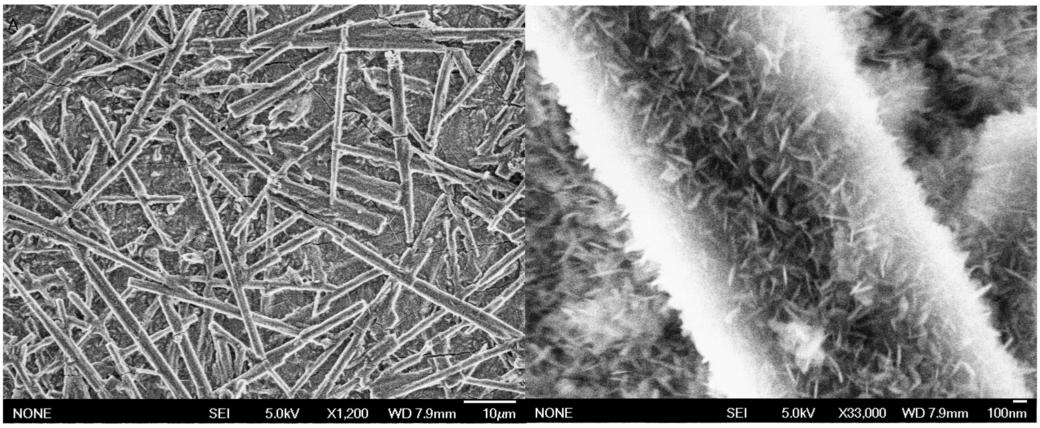

Figure S4 A and B SEM images of Ag nanoplates on nanowires with different magnification.

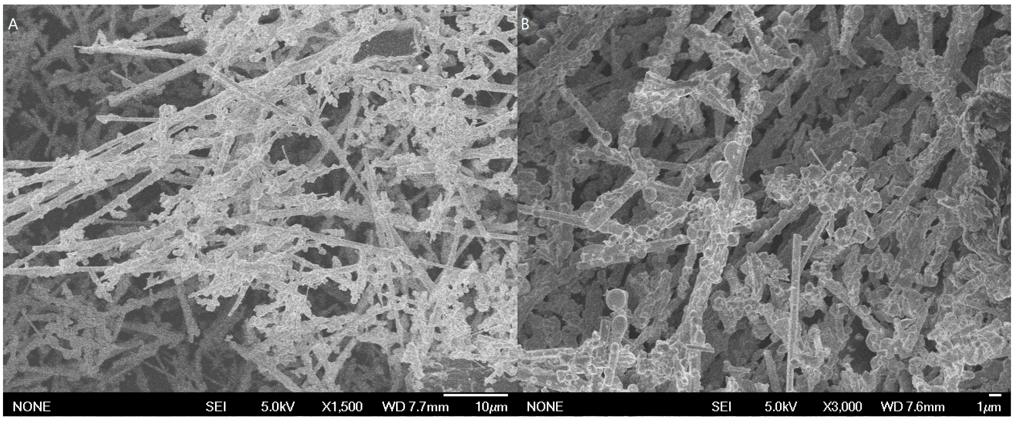

Figure S5. A and B SEM images of Ag nanoparticle on nanowires with different magnification.
